# To text or not to text? Acceptability of WeChat and text messaging intervention to promote tobacco control assistance among parents who smoke in rural China

**DOI:** 10.18332/tid/114089

**Published:** 2019-12-03

**Authors:** Xiaoxiao Chen, Duan Zhao, Tong Wen, Xia Xiao, Zixian Pan, Jingyi He, Pinpin Zheng, Wei Hao, Haijiang Lin, Abu S. Abdullah

**Affiliations:** 1 Taizhou City Center for Disease Control and Prevention, Taizhou, China; 2 Global Health Research Center, Duke Kunshan University, Kunshan, China; 3 School of Public Health, Kunming Medical University, Kunming, China; 4 Department of Preventive Medicine, School of Public Health, Fudan University, Shanghai, China; 5 Duke Global Health Institute, Duke University, Durham, United States; 6 Boston Medical Center, Boston University School of Medicine, Boston, United States

**Keywords:** tobacco control, WeChat, mobile health, text messaging, rural China

## Abstract

**INTRODUCTION:**

Although the use of mobile health (mHealth) to promote tobacco control and smoking cessation interventions has been available in developed countries, their use in low- and middle-income countries (i.e. in China) is lacking. This study examined the acceptance of text messaging and/or WeChat based tobacco control intervention among parents who smoke, in rural China.

**METHODS:**

Using a structured questionnaire, we surveyed smoker households (n=668) of children aged ≤5 years in two rural regions of southern China. Descriptive analyses were used to characterize respondents; multivariate regression analysis was used to test the associations between participants’ sociodemographic and other characteristics, and their acceptability of text messaging and/or WeChat intervention for tobacco control.

**RESULTS:**

After adjusting for other variables (i.e. region, ethnicity, age, education level, occupation, attitudes towards smoking, perception of cigarettes addictiveness, and quitting smoking), the overall knowledge about smoking and secondhand smoke (SHS) exposure significantly predicted a higher acceptance to text messaging/WeChat intervention (OR=0.567; 95% CI: 0.457–0.704). Participants who thought smoking made people feel relaxed were less likely to accept text messaging/WeChat intervention than those who did not think so (OR=1.403; 95% CI: 1.080–1.822).

**CONCLUSIONS:**

Our findings suggest that households in rural China that were more knowledgeable about the hazards of smoking and SHS exposure, and had negative feelings about the benefits of smoking, were more likely to accept text messaging/WeChat for tobacco control intervention. Understanding rural smokers’ preferences to receive intervention and related characteristics can help with the design of targeted tobacco control intervention programs in rural China.

## INTRODUCTION

Tobacco use is the leading preventable cause of avoidable morbidity and mortality in China^1^. China consumes approximately 40% of the world’s total cigarettes, and smoking caused about 20% of all adult male deaths in 2010^2,3^. With the current smoking rate, the annual number of tobacco deaths will reach 2 million by 2030, doubling from the 1 million deaths in 2010^3^.

At the same time, secondhand smoke (SHS) exposure is related to increased risk of lower respiratory tract infections, childhood cancers, asthma, allergies, and delayed physical and psychological development among children^4-6^. In China, about 58% to 73% of non-smoking pregnant women were exposed to SHS and the exposure rate remained at 41.9% after the babies were born^7,8^. SHS exposure prevalence among adult non-smokers in rural areas is also high, ranging from 62% to >70%^9,10^. The Chinese government, in recent years, has taken several preventive and policy measures to address smoking and reduce exposure of SHS to non-smokers and children^2^, including national bans on smoking in public and workplaces and tobacco advertising, and cessation support. However, these measures are not widely available or implemented in rural areas, which is due to the lack of enforcement of these policies in rural areas and the lack of available resources in rural areas. Also, interference by the tobacco industry for the wide implementation of tobacco control measures played a role^2^. The high SHS exposure and its associated risks together with the unavailability of policy measures underscore the importance of creating tobacco control interventions in rural areas.

Mobile health (mHealth) interventions have been shown effective in reducing smoking rates and have more advantages than traditional healthcare personnel-delivered intervention methods including its flexibility, accessibility, low cost, customizability, and scalability to large populations in some studies^11-14^. For example, in a recent meta-analysis of 13 randomized controlled trials, the text messaging intervention group had significantly higher quit smoking rates than the control group (OR=1.35; 95% CI: 1.23–1.48)^13^. Although mHealth intervention has shown to have some advantages in promoting health, a recent systematic review reported the limitations of mHealth interventions and that more research to assess the efficacy of mHealth interventions^15^ was warranted. Similarly, although the use of mHealth to promote population health has been increasing in China^16-18^, only a few studies examined the use of mHealth (i.e. text messaging or short message service, SMS) for tobacco control^19,20^ and no studies examined the potential to use mHealth in tobacco cessation among rural populations.

Considering the lack of tobacco control interventions in rural China and the growing evidence on the effectiveness of SMS-based interventions for quitting smoking in other countries, this study assessed the acceptance of text messaging intervention among parents who smoke in rural China. This study is a preparatory study for a pilot mHealth tobacco cessation intervention study using WeChat (a popular Chinese multi-purpose messaging, social media and mobile payment app used by 1.1 billion Chinese) and SMS to target the Chinese rural population. In the planned smoking cessation program, it is planned to use WeChat to deliver targeted tobacco control messages to rural smokers. Our specific aims for this study are to elucidate preferences for receiving smoking cessation information and support through text messages and whether sociodemographic (i.e. the region, ethnicity, age, occupation) or other behavioral factors (i.e. knowledge about smoking and SHS exposure influence) may influence one’s preference. Findings from this study could provide insights into developing WeChat or SMS-based tobacco use prevention and cessation intervention in rural China and other low- and middle-income countries (LMICs).

## METHODS

### Settings

The study settings were rural communities in Taizhou and Dali. Villages were purposively selected to cover diverse sociocultural aspects of the local population, as recommended by the local collaborators.

Taizhou is located in central Zhejiang province, the eastern part of China. Zhejiang is economically developed and the GDP per capita of Taizhou was ¥72912 (about US$10000) in 2017^21^. The rural population accounted for 37.8% of the total population (about 6 million) of Taizhou^21^. Four villages were purposively selected for this study, including two in Luqiao district and two in Linhai city.

Dali is the autonomous prefecture for the Bai minority, located in the western part of China. The GDP per capita in 2017 of Dali was ¥29846 (about US$4500)^22^. All the four villages from Jianchuan country were purposively selected for this study. The total population of Jianchuan was over 18 million in 2016, with ethnic Bai people accounting for the majority (89.9%)^23^.

### Sample

The study population consisted of household members of children aged ≤5 years in the selected community. To be eligible, all these households must have at least an adult smoker (smoking one or more cigarettes daily for the past 30 days) who resided in the same house with the child and were able to communicate in Mandarin Chinese. All the eligible households were approached to participate in the study. These households were part of another large ongoing study addressing secondhand smoke exposure among children. One smoker and the main caregiver of the child were invited to participate in the survey. If a household contained more than one smoker, then the smoker who spends more time with the child was recruited.

### Procedure

Local community health workers (CHWs) or village doctors screened households, following the child health records available in the community health center, and invited eligible households to participate in the study. Trained interviewers, accompanied by CHWs, visited households that agreed to participate in the study during a pre-agreed time. The interviewers described the purpose and details of the study and sought informed consent from the participants. After confirming subjects’ informed consent, interviewers completed the survey interview. The study was reviewed and approved by the Ethics Committee of Duke Kunshan University (IRB No: 2016ABDU003).

### Data collection

The study was conducted in March 2018 in Taizhou and June 2018 in Dali. Students from local colleges/universities were recruited as interviewers and trained to collect data. The interviewers received a half-day training (3 hours), delivered by the research team, to learn the necessary communication and interviewing skills, ethical conduct of human subject’s research, and details of the study. One of the researchers (JH) took the interviewers through the questionnaires question-by-question and explained the information required thoroughly. Mock interview sessions were performed to allow interviewers to practice.

A structured Chinese questionnaire was used for data collection. The questionnaire contained two parts, Part I for smokers and Part II for caregivers. If the smoker was the same person as the caregiver, then he/she only needed to answer the questions about the children’s health in Part II. The questionnaire was either filled in by either the subjects themselves or the subjects responded to the questions orally and the interviewers wrote down the answers on their behalf. The data used and analyzed in this study are available from the corresponding author upon reasonable request.

### Measures

In this study, two questionnaires were used for smokers and caregivers, separately. The domains of the questionnaire included the following.

#### Sociodemographic characteristics

These included: region, type of participants (smoker or caregiver), gender, age, education background, ethnicity, marital status, and occupation.

#### Knowledge about smoking and SHS exposure

This was measured by asking 8 items (Table 2). The first four items were measured using a 5-point scale with response categories ranging from 1 (minimum risk) to 5 (maximum risk). The last four items were measured using a 5-point scale with response categories ranging from 1 (totally disagree) to 5 (totally agree) with those responding ‘I don’t know’ considered as ‘zero’. Higher scores indicated a higher level of risk cognition on each item. The mean total score for the 8 items was calculated to evaluate overall smoking-related risk cognition.

#### Attitudes towards smoking

These included 5 items (Table 2) and measured to what extent smokers believe the potential benefit of smoking. A 5-point scale was used with response categories ranging from 1 (totally disagree) to 5 (totally agree).

#### Attitudes towards quitting smoking

These were measured using 4 items (Table 2). A 5-point scale was used with response categories of 1 (totally disagree) to 5 (totally agree). For the item ‘quitting smoking can save a fortune’, a reverse of 5-point scale with responses ranging from 5 (totally disagree) to 1 (totally agree) was applied to calculate scores.

#### Perception of cigarette addictiveness

This was measured by asking a single question (Table 2), using a 5-point scale with response categories of 1 (totally disagree) to 5 (totally agree).

### Data analysis

Descriptive statistical analyses were conducted for sociodemographic variables and scores of related characteristics of participants. Means with standard deviation (SD) were calculated for quantitative data, while percentages were calculated for qualitative data. Student’s t-test was used to compare characteristics differences between participants who would and would not accept text messaging or WeChat for tobacco control assistance intervention. Chi-squared and Fisher exact probability tests were used to compare the acceptability between different sociodemographic groups. Statistically significant sociodemographic variables and related characteristics of participants by acceptability were included in the multivariate logistic regression analysis. Hosmer-Lemeshow goodness-offit test was conducted to test how well the logistic regression model fits the data and to reject the null hypothesis of adequate fit when p<0.05. All collected data were analyzed with SAS 9.4 (SAS Institute, Inc., NC). The level of p<0.05 was considered statistically significant in all tests.

## RESULTS

Of the 1017 households approached (307 in Dali and 710 in Taizhou), 66% (668/1017) agreed to participate, 12% (121/1017) did not meet the inclusion criteria, and 23% (232/1017) refused to participate. The reasons for refusal were: not interested in a tobacco control study (n=154), and no time (n=78). Overall, a total of 668 smokers (206 from Dali and 462 from Taizhou) were available for the analyses. All of the participants had a mobile phone with free access to SMS. Smokers consumed an average of 16 cigarettes per day (95% CI: 15–17). Of the total number of subjects, almost all (99%) smoked cigarettes and the remaining 1% smoked e-cigarettes or other tobacco products. As shown in Table 1, most of the participants were male (99%) and married (99%). The mean age was 43.4 (±13.75) years with a range 20–77 years. Among the participants, 30.03% attained education to high school or above level and nearly half (47%) attained education to middle school. With regard to occupation, 35% of participants were farmers and 21% were workers. There are statistical differences in several sociodemographic factors (i.e. region, age group, ethnicity, and education) between participants who would accept text message/WeChat based intervention and those who would not accept (Table 1).

**Table 1:** Demographic characteristics of participants by acceptability to text messaging or WeChat for tobacco control assistance intervention (N=668)

*Demographics*	*Overall*	*Accept SMS and/or WeChat*	*Do not Accept SMS and/or WeChat*	*p*

	*n (%)*	*n (%)*	*n (%)*	
**Region** (n=668)				**0.0308**
Taizhou	462 (69)	246 (57)	186 (43)	
Dali	206 (31)	132 (66)	68 (34)	
**Age** (n=668)				**0.0329**
≤34	247 (37)	148 (63)	86 (37)	
35–54	227 (34)	134 (63)	79 (37)	
≥55 years	194 (29)	96 (52)	89 (48)	
**Gender** (n=665)				0.0753*
Male	659 (99)	372 (59)	254 (41)	
Female	6 (1)	5 (83)	1 (17)	
**Ethnicity** (n=662)				**0.0085**
Chinese Han	474 (72)	252 (57)	193 (43)	
Minority	188 (28)	125 (68)	59 (32)	
**Marital status** (n=627)				0.0773*
Married	619 (99)	373 (60)	246 (40)	
Single	7 (1)	3 (43)	4 (57)	
Divorced or widowed	1 (0)	0 (0)	1 (100)	
**Occupation** (n=663)				0.0687
Farmer	233 (35)	136 (62)	84 (38)	
Worker	177 (27)	96 (57)	72 (43)	
Self-employment	138 (21)	83 (64)	46 (36)	
Unemployed	41 (6)	16 (40)	24 (60)	
Other	74 (11)	45 (63)	27 (37)	
**Education** (n=656)				**0.0392**
Primary school or below	152 (23)	75 (52)	70 (48)	
Middle school	307 (47)	188 (64)	104 (36)	
High school or above	197 (30)	111 (60)	74 (40)	

* Fisher exact probability test.

### Characteristics of participants by acceptability to text messaging or WeChat intervention

The mean total score of knowledge about smoking and SHS exposure was 3.82±0.93. Participants who were more likely to accept text messaging/WeChat intervention had a higher score of knowledge about smoking and SHS exposure than participants who would not accept (p<0.0001). There were significant differences in the mean score of all items for knowledge about smoking and SHS exposure between those who will accept and will not accept text messaging/WeChat. For attitudes towards smoking, participants who were less likely to accept text messaging/WeChat intervention had a higher score on the item of ‘smoking makes people feel relaxed’ (p=0.0092) and the item of ‘smoking can help people focus and think’ than the participants who did not think so. For perception of cigarettes addictiveness, participants who were more likely to accept text messaging/WeChat intervention held a more negative attitude towards cigarettes addictiveness (p=0.0058) than those who will not accept. For quitting smoking, participants who were less likely to accept text messaging/WeChat intervention had a higher score on the item ‘quitting smoking can make people lose joy in their lives and have nothing to fall back on’ (p=0.0427) (Table 2).

**Table 2:** Related characteristics of participants by acceptability to text messaging or WeChat for tobacco control assistance intervention (N=668)

*Variables*	*‘I don’t know’*	*Overall*	*Accept SMS and/or WeChat*	*Do not Accept SMS and/or WeChat*	p*

	*n (%)*	*Mean ± SD*	*Mean ± SD*	*Mean ± SD*	
**Knowledge about smoking and SHS exposure**					
Smoking increases the risk of lung cancer	-	4.07±1.16	4.21±1.03	3.82±1.30	**<0.0001**
Smoking increases the risk of COPD	-	4.00±1.16	4.17±1.04	3.72±1.29	**<0.0001**
Smoking increases the risk of heart diseases	-	3.65±1.34	3.84±1.23	3.33±1.42	**<0.0001**
Concerned about the harmful effects of SHS exposure to children’s health	-	4.19±1.13	4.39±0.97	3.89±1.30	**<0.0001**
Exposure to SHS can cause lung cancer in non-smokers	126 (18.9)	2.93±1.68	3.13±1.66	2.64±1.67	**0.0004**
Exposure to SHS is harmful to infant and children’s health	28 (4.2)	3.91±1.19	4.08±0.98	3.66±1.41	**<0.0001**
Exposure to SHS is harmful to adults’ health	38 (5.7)	3.67±1.27	3.89±1.07	3.35±1.47	**<0.0001**
Smoking around your children would cause adverse health effects among children	31 (4.6)	3.91±1.20	4.11±0.97	3.60±1.43	**<0.0001**
The mean total score of knowledge about smoking and SHS exposure	-	3.82±0.93	4.02±0.79	3.51±1.04	**<0.0001**
**Attitudes towards smoking**					
Smoking makes people feel energetic	-	3.36±1.04	3.34±1.02	3.45±1.05	0.2078
Smoking makes people feel relaxed	-	3.54±0.97	3.48±0.98	3.69±0.93	**0.0092**
Smoking can help people focus and think	-	3.42±0.99	3.37±1.01	3.53±0.94	**0.0464**
Smoking is beneficial for social interaction	-	3.52±1.04	3.48±1.06	3.59±0.99	0.2048
Smoking can be a self-controlled behavior	-	3.08±1.09	3.10±1.08	3.03±1.12	0.4156
Mean total score of attitudes towards smoking	-	3.39±0.71	3.36±0.73	3.47±0.66	0.2157
**Perception of cigarette addictiveness**					
If I smoke cigarettes, I may become addicted	-	3.67±1.01	3.77±0.93	3.54±1.09	**0.0058**
**Quitting smoking**					
Quitting smoking can save a fortune	-	2.02±0.95	1.97±0.91	2.10±1.00	0.0971
Quitting smoking can make people lose joy in their lives and have nothing to fall back on	-	2.83±1.07	2.76±1.05	2.94±1.09	**0.0427**
Quitting smoking may lead to other health problems, such as weight gain	-	3.22±1.05	3.23±1.06	3.18±1.06	0.5553
Quitting smoking may affect smokers’ work or quality of life	-	2.83±1.06	2.80±1.08	2.87±1.06	0.4477
Mean total score of quitting smoking		2.72±0.66	2.69±0.65	2.77±0.69	0.1403

* For Student’s t-tests.

### Predictors of acceptance to text messaging and/or WeChat based intervention among participants

In a multivariate analysis, after adjusting for other variables (i.e. region, ethnicity, age, education level, occupation, attitudes towards smoking, perception of cigarettes addictiveness and quitting smoking), the overall knowledge about smoking and SHS exposure significantly predicted a higher acceptance of text messaging/WeChat based intervention (OR=0.567; 95% CI: 0.457–0.704). Participants who thought smoking made people feel relaxed were less likely to accept text messaging/WeChat intervention than participants who did not think so (OR=1.403; 95%CI: 1.080–1.822) (Table 3).

**Table 3:** Multivariate analysis of acceptance to text messaging or WeChat for tobacco control intervention among smokers in rural China (No vs Yes)

*Factors*	OR*	*95% CI*
**Region**		
Dali vs Taizhou	1.809	0.575–5.687
**Ethnicity**		
Minority vs Chinese Han	0.440	0.137–1.410
**Age** (years)		
34–54 vs <34	0.933	0.599–1.454
≥55 vs <34	1.273	0.748–2.168
**Education**		
Middle school vs Primary school or below	0.942	0.559–1.589
High school or above vs Primary school or below	1.358	0.750–2.460
**Occupation**		
Worker vs unemployed	0.738	0.331–1.643
Self-employed vs unemployed	0.682	0.294–1.582
Farmer vs unemployed	0.649	0.302–1.397
Other vs unemployed	0.570	0.230–1.414
**Knowledge about smoking and SHS exposure**		
Mean total score	**0.567**	**0.457–0.704**
**Attitudes towards smoking**		
Smoking makes people feel relaxed	**1.403**	**1.080–1.822**
Smoking can help people focus and think	0.970	0.759–1.238
**Perception of cigarette addictiveness**		
If I smoke, I may become addicted	0.831	0.687–1.005
**Quitting smoking**		
Quitting smoking can make people lose joy in their lives and have nothing to fall back on	1.056	0.882–1.264

The logistic regression model is statistically significant (p<0.0001).

* Adjusting for other variables (i.e. region, ethnicity, age, education level, occupation, attitudes towards smoking, perception of cigarettes addictiveness and quitting smoking).

## DISCUSSION

To our knowledge, this is the first study that explored WeChat/text messaging intervention preferences and associated characteristics to inform future smoking cessation intervention implementation using mHealth tools, in rural China. Preferences-related factors including region, age, gender, ethnicity, marital, occupation, and education status were explored.

The findings show that more than half (56.5%) of our participants will accept text messaging and/or WeChat for tobacco control messaging. Though we found the acceptance of text messaging intervention is a little lower (52%) in the age group ≥55 years than the other two younger age groups of 34–54 and <34 (63%) years, and among unemployed (40%) than other occupational groups (57–64%), the differences were not statistically significant. Our results contradict the findings of previous studies that found that age and occupation were strongly associated with acceptance of mHealth interventions in Bangladesh^24^ and China^25^. The general acceptance among young people is understandable as they are more familiar with digital devices, as technologies are ubiquitous in their lives, while the elderly are often slower in learning new technologies and more skeptical about the use of technologies^26^. Our results might be explained by the fact that the mobile phone use rate is very high in China and participants were accustomed to mobile phone use despite their age group; WeChat had an overall usage rate of 94.5% among Chinese citizens in March 2018^27^.

In this study, we were unable to measure the acceptance of mHealth approach among female smokers in the rural area due to the low smoking rate among females in China^28^, and the small number of female smokers we had in the current study (n=6). However, 5 out of the 6 females will accept text messaging/WeChat based intervention underscoring acceptance of text messaging among females. In earlier studies, females were less likely to accept the mHealth approach than males in China^28,29^. In general, men are more task-orientated and are more interested in technology, therefore, men are more likely to accept mHealth^29,30^.

Consistent with the findings of an earlier study that did not find any association between educational attainments and acceptance of the smoking cessation education app^31^, we also did not find any association between acceptance of text messaging and educational level. This might be attributable to the fact that mobile phone and WeChat use is so high in China, that it does not vary among people with different levels of education^27^.

In this study, we found a strong association between knowledge about smoking and SHS exposure and acceptance of text messaging/WeChat based intervention. The more smokers realize the harm of tobacco use, the more likely they would accept text messaging/WeChat intervention approaches. Similarly, those who believed tobacco use has a positive effect, in making people feel relaxed, were less likely to accept text messaging/WeChat intervention. In earlier studies^32-35^, a higher level of smoking-related knowledge was associated with favorable attitudes towards quitting smoking. We also have found similar findings in the current study. This finding underscores the importance of educational campaigns to increase people’s awareness and knowledge about smoking and SHS exposure and the benefits of quitting smoking to promote the acceptance of mHealth based intervention among smokers in rural areas. The higher acceptance of mHealth based intervention (56.5% in the current study) among the rural Chinese underscores the possibility of delivering evidence-based tobacco control information to the rural public using mHealth tools.

To increase the effectiveness of mHealth interventions, we should take advantage of the versatility of WeChat and explore new ways to promote educational interventions. For example, WeChat has already established a sophisticated advertisement platform that could deliver information to different target populations based on their age, gender, education levels, locations, marital status, interests, and behaviors (including health-related), or even customized to a population using their phone numbers^36^. Therefore, it is possible to tailor smoking cessation information via advertising format in ‘moments (functioning like Facebook)’, ‘subscription articles’, and ‘mini-program’. It is also possible to customize the delivery of the intervention to smokers with a low level of education in the future via WeChat. Future studies that would use WeChat to deliver interventions should explore these innovative approaches to increase intervention effectiveness.

### Limitations

There are a few limitations to this study. First, our study considered age, gender, ethnicity, education, and knowledge and attitudes toward smoking as moderator variables; some other variables may also affect people’s willingness to accept mHealth methods, e.g. concern about privacy and cell phone use pattern. The low trust in the personalization of services may also negatively affect one’s acceptance of text messaging or WeChat message^25^. People with higher purchasing power may have a higher acceptance of mHealth based intervention^24^. Second, this research explored only the perceived acceptance (i.e. intention) rather than the actual usage of a text message or WeChat services for tobacco control assistance. Thus, the actual acceptance rate should be addressed in future research. Third, it is not clear whether the preference of our participants to receive text messaging-based intervention could be generalizable to other rural areas in China because both selected study areas are in southern China. However, the sample demographics included a coastal rural area (affluent) and an inland rural area (less affluent), two diverse socioeconomic groups of Chinese rural smokers, so we expect our findings can be generalized to this important target population in other regions of China.

## CONCLUSIONS

Overall, this study provides important information regarding the acceptance of text messaging and/ or WeChat for tobacco control intervention among rural Chinese. Our study found that smokers in rural areas with more knowledge about smoking and SHS exposure were more likely to accept tobacco control intervention delivered via mHealth tools, including text messaging and WeChat message. At the same time, we found that having a favorable attitude towards smoking was negatively associated with accepting text messaging intervention on tobacco control assistance. To address smoking-related health disparities and to promote tobacco control in rural China, future mHealth based public health programs should consider rural smokers’ preferences and characteristics as identified in the current study.
